# Waste Soybean Oil and Corn Steep Liquor as Economic Substrates for Bioemulsifier and Biodiesel Production by *Candida lipolytica* UCP 0998

**DOI:** 10.3390/ijms17101608

**Published:** 2016-09-23

**Authors:** Adriana Ferreira Souza, Dayana M. Rodriguez, Daylin R. Ribeaux, Marcos A. C. Luna, Thayse A. Lima e Silva, Rosileide F. Silva Andrade, Norma B. Gusmão, Galba M. Campos-Takaki

**Affiliations:** 1 Fungal Biology Post-graduation Program, Federal University of Pernambuco, 50670-901 Recife-PE, Brazil; adrife.souza@gmail.com; 2 Nucleus of Research in Environmental Sciences and Biotechnology, Catholic University of Pernambuco, 50050-590 Recife-PE, Brazil; dayanamontero87@gmail.com (D.M.R.); drubioribeaux@gmail.com (D.R.R.); macluna@bol.com.br (M.A.C.L.); ithalv@yahoo.com.br (T.A.L.e.S.); rosileide_fontenele@yahoo.com.br (R.F.S.A.); 3 Biological Sciences Post-graduation Program, Federal University of Pernambuco, 50670-420 Recife-PE, Brazil; 4 Northeast Network for Biotechnology Post-graduation Program, Federal Rural University of Pernambuco, 52171-900 Recife-PE, Brazil; 5 National Post-Doctorate Program-CAPES, Catholic University of Pernambuco, 50050-900 Recife-PE, Brazil; 6 Department of Antibiotics, Federal University of Pernambuco, 50670-901 Recife-PE, Brazil; normagusmao@gmail.com

**Keywords:** *Candida lipolytica*, agro-industrial waste, bioemulsifier, renewable sources, lipids, biofuel

## Abstract

Almost all oleaginous microorganisms are available for biodiesel production, and for the mechanism of oil accumulation, which is what makes a microbial approach economically competitive. This study investigated the potential that the yeast *Candida lipolytica* UCP0988, in an anamorphous state, has to produce simultaneously a bioemulsifier and to accumulate lipids using inexpensive and alternative substrates. Cultivation was carried out using waste soybean oil and corn steep liquor in accordance with 2^2^ experimental designs with 1% inoculums (10^7^ cells/mL). The bioemulsifier was produced in the cell-free metabolic liquid in the late exponential phase (96 h), at Assay 4 (corn steep liquor 5% and waste soybean oil 8%), with 6.704 UEA, IE_24_ of 96.66%, and showed an anionic profile. The emulsion formed consisted of compact small and stable droplets (size 0.2–5 µm), stable at all temperatures, at pH 2 and 4, and 2% salinity, and showed an ability to remove 93.74% of diesel oil from sand. The displacement oil (ODA) showed 45.34 cm^2^ of dispersion (central point of the factorial design). The biomass obtained from Assay 4 was able to accumulate lipids of 0.425 g/g biomass (corresponding to 42.5%), which consisted of Palmitic acid (28.4%), Stearic acid (7.7%), Oleic acid (42.8%), Linoleic acid (19.0%), and γ-Linolenic acid (2.1%). The results showed the ability of *C. lipopytica* to produce both bioemulsifier and biodiesel using the metabolic conversion of waste soybean oil and corn steep liquor, which are economic renewable sources.

## 1. Introduction

Biosurfactants are amphipathic molecules with different degrees of polarity, which means they can be used to reduce surface tension, and to form emulsions where the hydrocarbon can be solubilized in water and is thus called a bioemulsifier [1,2].

A variety of microorganisms synthesize into extracellular environmental amphiphilic compounds, such as biosurfactants and bioemulsifiers. These have several advantages over synthetic compounds, the most important of which is their biodegradability because this avoids problems of toxicity and accumulation in natural ecosystems [3].

The terms biosurfactants and bioemulsifiers have been used with reference to the same level of bioactivity. However, compounds of high molecular weight and capable of forming and stabilizing emulsions are referred to as bioemulsifiers or bioemulsans. These are polymers of polysaccharides, lipo-polysaccharides, proteins and lipoproteins while biosurfactants are represented by compounds of low molecular weight, amongst which lipopeptides and glycolipids are included [2,3,4].

*Candida lipolytica* is considered an oleaginous yeast that can accumulate a proportion of 40% of intracellular lipids. Moreover, it has the ability to grow on a wide variety of hydrophobic substrates due to its adjustment because gene-encoding surfactants (biosurfactants) evolve which act as detergents and help add to the availability of these insoluble substrates by forming emulsions [5]. Liposan is one of the most studied bioemulsifiers produced by this single-celled fungus [6,7,8]. Lipids produced by this microorganism have chemical and energy values similar to those of vegetable and animal oils [9,10].

A further area that has received considerable attention in recent years is how best to use polysaccharide–protein electrostatic complexes as emulsifiers. The stabilization of the emulsions is related to adsorbed protein molecules because they prevent droplets aggregating and because they coalesce as a result of electrostatic and/or steric repulsive forces. The process has limitations and droplet aggregation will occur whenever the protein isoelectric point has a net zero charge and unless the adsorbed layer thickness is large enough [11,12,13].

Making use of oleaginous fungi to produce biodiesel is limited, although these fungi have several advantages over conventional sources such as plants and algae. They can be easily grown in bioreactors, have short life cycles, exhibit rapid growth, and do not need much space. Light or climatic variations are easier to handle and these fungi can use a wide variety of low-cost energy sources, such as lignocellulosic biomass and agricultural residues [14].

Thus, this set out to evaluate: (a) the potential of *Candida lipolytica* UCP0988 when producing (extracellular metabolite) bioemulsifiers and emulsification activity; and (b) the light microscopy characteristics of the droplets’ requirements for stable emulsification. It also set out to assess biodiesel (from biomass), as an economic, competitive and promising bioprocess since it uses inexpensive substrates such as waste soybean oil and corn steep liquor as carbon and nitrogen sources.

## 2. Results

### 2.1. Production of Bio-Emulsifier by Candida lipolytica UCP 0988 Using Agro-Industrial Waste

*Candida lipolytica* demonstrated an ability to produce bio-emulsifier in medium containing 5% corn steep liquor and 8% waste soybean oil on Assay 4 of the factorial design (Table 1). It was in this condition that the highest emulsification index (96.66%) was obtained with burned engine oil after 96 h of cultivation, whereas the maximum emulsification activity obtained was 6.704 UEA with n-hexadecane, and was characterized as anionic profile by a Zeta meter.

**Figure 1:**
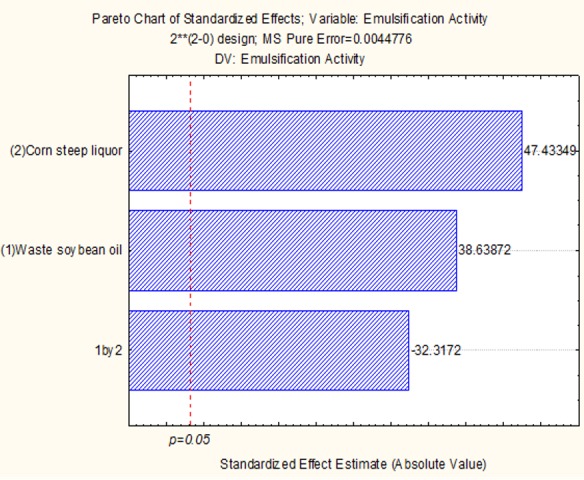
Pareto chart for 2^2^ factorial design of culture after 96 h, where (1) corn steep liquor and (2) waste soybean oil are the independent variables and emulsifying activity is the response variable. The point at which the effect estimates were statistically significant is shown.

The statistical analyses for emulsification activity using n-hexadecane showed by Pareto chart the standardized effects (Figure 1). Figure 1 demonstrated that the independent variables were positively significant. Corn steep liquor substrate was of the first order of significance, but in the interactions of both waste soybean oil and corn steep liquor, negative variables were observed and influenced the emulsifier activity of the variable response.

### 2.2. Characterization of Emulsion Droplets by Light Microscopy

**Figure 2:**
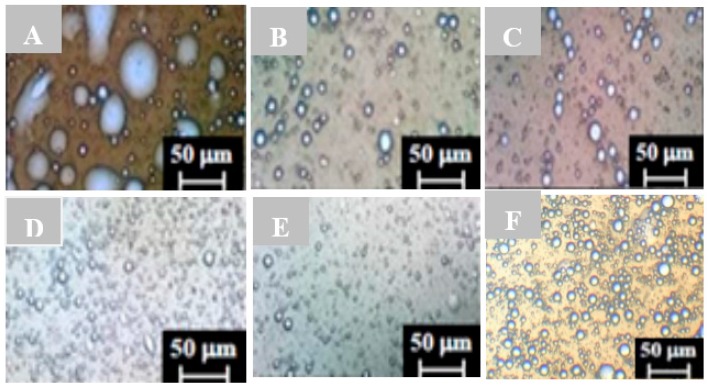
Comparison with the microstructural characteristics of the emulsions droplets formed from bioemulsifier produced by *C. lipolytica* UCP 0988, Assay 4 of the factorial design 2^2^ (5% (*v*/*v*) corn steep liquor and 8% (*v*/*v*) waste soybean oil) using light microscopy: (**A**) liquid without inoculum; (**B**) culture at 24 h; (**C**) culture at 48 h; (**D**) culture at 72 h; (**E**) culture at 96 h; and (**F**) Control (Sodium dodecyl sulfate-SDS).

Emulsions were formed using burned engine oil as the hydrophobic substrate in the best condition (4) of the factorial design (5% corn steep liquor and 8% waste soybean oil) (Figure 2). The comparison of droplets by studying the stability of the emulsion was initiated from the liquid medium as control. The droplets formed are large, but are not globular in appearance as a result of the coalescence formed (Figure 2A). However, the use of the metabolic liquid after 24 h of fermentation formed droplets that were clearly globular in appearance. Due to poly-dispersion and flocculation, they tended to coalesce to larger volumes (Figure 2B). In addition, free spaces in the liquid phase were generated. In the emulsion containing the liquid medium, few spaces between the droplets were observed, but the presence of films encompassing the oily phase of the smaller droplets was seen (Figure 2C). In the emulsion formed after 72 h of fermentation, different aspects of droplets were observed. For example, some were smaller (Figure 2D). However, in the emulsions using metabolic liquid after 96 h, smaller droplets were seen which exhibited homogeneity between phases, and greater stability (Figure 2E). Figure 2F showed emulsion control using the anionic chemical surfactant (sodium-dodecyl sulfate (SDS)), and droplets of varying sizes (medium to large), few empty spaces and homogeneity between the phases and greater stability were observed.

Table 2 shows the average diameter of the droplets, the emulsification index and activity, and properties of the emulsification. The emulsion droplets varied in size (30–0.1 μm), generating a distribution of droplet sizes during the growth and bioemulsifier production by *C. lipolytica*. The formation of smaller emulsion droplets (0.1 to 5 μm) was noticeable in the last hours of the fermentation. The comparison between the distribution of the size of particles and the emulsification index (E24) and Units of Emulsification Activity (UEA) increased when the bioemulsifier was produced during the stationary phase (96 h), and the results of the droplets size area and stability are influenced by the maximum of anionic bioemulsifier production. The comparison of the control with anionic biosurfactant SDS showed similar behavior as well the bioemulsifier produced by *Candida lipolytica*.

### 2.3. Stability of Bioemulsifier

**Figure 3:**
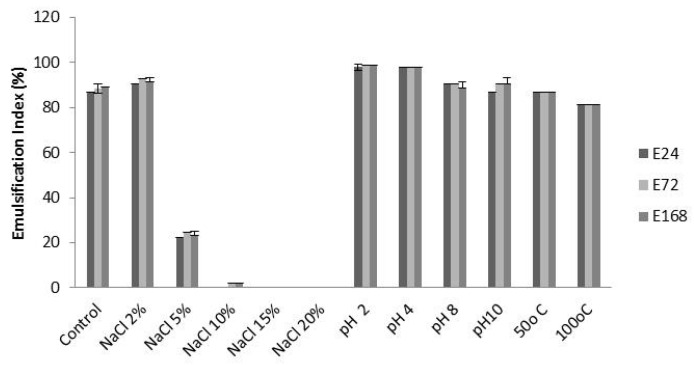
Emulsification index of bioemulsifier produced by *Candida lipolytica* UCP 0988 against burnt engine oil when NaCl concentrations, pH and temperature were varied.

This study analyzes the influence of these factors on the stability of bioemulsifier produced by *C. lipolytica* in Condition 4 of the factorial design, with metabolic liquid, after 24, 72 and 168 h of emulsion (Figure 3). The bioemulsifier produced by *Candida lipolytica* in the conditions examined in this paper, on using burned engine oil as a hydrophobic substrate, showed that its emulsifying stability was maintained when 2% NaCl was added. In concentrations above this amount, the bioemulsifier was inactive. On the other hand, after the metabolic liquid had been subjected to varied pH, it was observed that the bioemulsifier maintained its properties in all pH levels tested, there being a maximum percentage of emulsions (100%) in pH 2 and 4 that lasted for seven days. Additionally, the bioemulsifier maintained stability in both both temperatures tested (50 and 100 °C), resulting in emulsification values of 85% and 90%, respectively.

### 2.4. Potential of Bioemulsifier on Displacement of Hydrophobic Compound in Seawater

**Figure 4:**
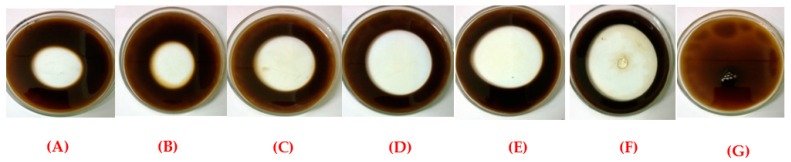
Dispersion of burnt engine oil in seawater by bioemulsifier produced by *C. lipolytica* UCP 0988 in accordance with the factorial design: (**A**) Assay 1; (**B**) Assay 2; (**C**) Assay 3; (**D**) Assay 4; (**E**) central point; (**F**) SDS; and (**G**) water.

In this study, the oil dispersion test was performed to evaluate the ability of bioemulsifier to disperse hydrophobic substrates in all conditions of the factorial design (Figure 4). The bioemulsifier of *C. lipolytica* showed significant dispersant capacity after 96 h in Assay 1 (Figure 4A), 2 (Figure 4B) and 3 (Figure 4C), resulting in dispersions of 12.56, 14.13 and 20.41 cm^2^, respectively. In addition, in Test 4 (Figure 4D), the bioemulsifier showed a higher oil dispersion capacity (38.46 cm^2^). However, the best results were observed at the central points of the factorial design (Figure 4E) resulting in dispersion with values between 38.46–45.34 cm^2^. SDS was used as positive control (Figure 4F), and water as the negative control (Figure 4G).

### 2.5. Application of Bioemulsifier to Remove a Hydrophobic Compound

The results showed for the removal of diesel oil pollutant adsorbed in the sand ealuated using cell-free culture medium from *C. lipolytica* (crude bioemulsifier) obtained after 96 h, showed removal of 93.74% of the hydrophobic pollutant. However, the use of distilled water as control removed only 18.14%. The tests were carried out using bioemulsifier cell free broth and the results obtained were considered satisfactory.

### 2.6. Production of Biomass and Total Lipids by Candida lipolytica UCP 0988 Using Agro-Industrial Waste

The maximum yield of biomass production by *C. lipolytica* was in Assay 4 of the factorial design (5% corn steep liquor and 8% waste soybean oil with 12.71 g/L) (Table 3). It was observed that the maximum concentrations of corn steep liquor and waste soybean oil in the production medium favored the high production of biomass.

**Figure 5:**
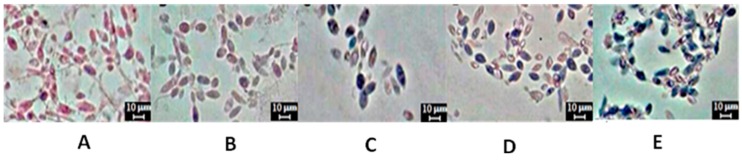
Cytochemistry staining of cells of *Candida lipolytica* UCP 0988 by Sudan Black method. Assay 4 of 2^2^ factorial design: (**A**) 0 and (**B**) 24 h no lipid accumulation ; (**C**) 48 h and (**D**) 72 h low lipid accumulation; and (**E**) 96 h higher lipid accumulation.

The microorganism accumulated percentages exceeded 36%. The best result achieved was 42% in Assay 4 (5% corn steep liquor and 8% waste soybean oil). Figure 5 shows the total lipid content accumulated in cells from 0 to 96 h in Assay 4 by cytochemical analysis using Sudan Black staining, thereby revealing the presence of lipid bodies of dark color. Young cells of *C. lipolytica* (Figure 5A) were used as controls to identify the accumulation of lipids. The results obtained in the first 24 h of culture (Figure 5B) showed there was no significant accumulation of lipids. After 48 h (Figure 5C) and 72 h (Figure 5D), a significant accumulation of lipids in most *C. lipolytica* cells occurred. However, the maximum accumulation of lipids occurred at 96 h (Figure 5E).

**Figure 6:**
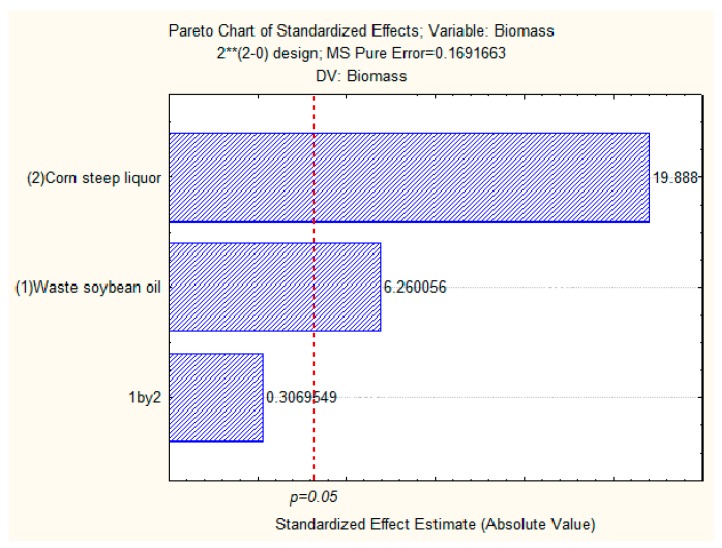
Pareto chart for 2^2^ factorial design of culture of 96 h, where (1) corn steep liquor and (2) waste soybean oil are the independent variables and biomass is the response variable. The point at which the estimates of the effect were statistically significant (*p* = 0.05) is indicated by the dotted red vertical line.

The evaluation of the concentrations of corn steep liquor and waste soybean oil, as well as their interactions when producing biomass by *Candida lipolytica* are represented by the Pareto diagram with 95% confidence as shown in Figure 6. According to statistical analysis, the independent variable, corn steep liquor, influenced the increase in the production of biomass more than the other independent variable, waste soybean oil. However, the association between both residues was not significant from a statistical point of view. As can be seen in the Pareto diagram, corn steep liquor and waste soybean oil positively influenced, with statistical significance, the increase in biomass production.

**Figure 7:**
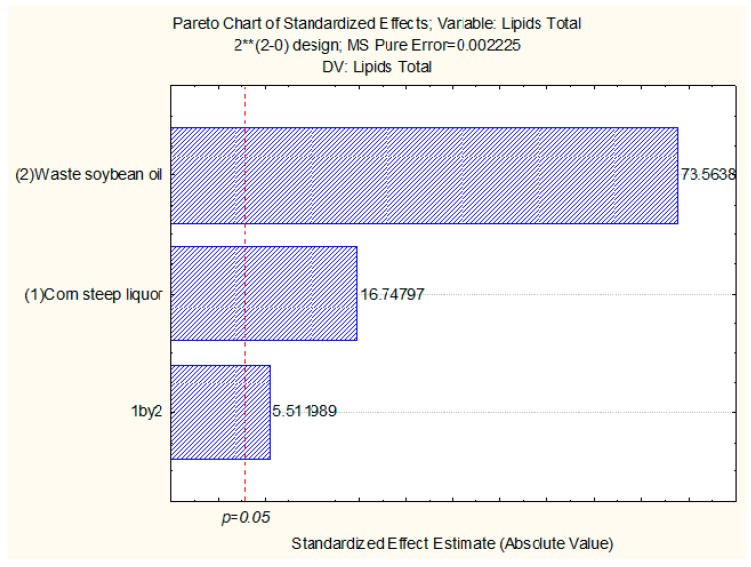
Pareto chart for 2^2^ factorial design of culture of 96 h, where (1) corn steep liquor and (2) waste soybean oil are the independent variables and the total of lipids is the response variable. The point at which the effect estimates were statistically significant (*p* = 0.05) is indicated by the dotted red vertical line.

According to statistical analysis, the independent variable, waste soybean oil, influenced the increase in the total production of lipids more than the other independent variable, corn steep liquor. As can be seen in the Pareto diagram, waste soybean oil and corn steep liquor had a statistically significant influence on the increase in the production of lipids (Figure 7).

### 2.7. Fatty Acid Profile of Total Lipids

The analysis of the fatty acid profile (type and concentration) is important for the conservation and oxidation of biodiesel, so it is of great importance to know what raw material should be used. In this study, the direct methylation of the biomass was performed to obtain fatty acid methyl esters (FAMEs) from the sample with the highest lipid yield (Assay 4), for the qualitative analysis of fatty acids of lipid bodies produced by the microorganism. The results, shown in Table 4, exhibit major amounts of oleic, palmitic and linoleic acid (Table 4). The profile obtained after cultivation in Condition 4 (5% corn steep liquor and 8% waste soybean oil) was 36.1% saturated fatty acids, 42.8% monounsaturated fatty acids and 21.1% polyunsaturated fatty acids.

### 2.8. Transesterification/Direct Esterification of Biomass

The simultaneous method of the extraction and transesterification/esterification of biomass ensures the complete reaction of lipids in methyl esters of fatty acids, including free fatty acids present in the total lipids, which is an important factor that determines the acidity of biodiesel.

In this study, the percentage of lipids of biomass produced in Condition 4 was 39% from which 37.9% biodiesel was obtained. Thus, a rate of approximately 96.70% of total lipids was transesterified, while only saponifiable lipids (triacylglycerides, dialcilglicerídes, monoalcilglicerídes and polar lipids) and free fatty acids are converted.

## 3. Discussion

### 3.1. Production of Bioemulsifier by Candida lipolytica UCP 0988 Using Agro-Industrial Waste

According to Willumsen and Karlson [15], values above 50% emulsification are considered significant. These data are promising given that the biotechnological production of bioemulsifiers depends on developing cheaper processes, which will make this process economically viable, since the culture medium is about 50% of the current final cost of the product [16].

Corn steep liquor has been shown to be a promising source when growing microorganisms, and has been included in media that are used to produce bioemulsifiers/biosurfactants [17,18,19].

As in this study, Almeida et al. [20] used residual soybean oil and corn steep liquor to produce biosurfactant by *Candida tropicalis* and using factorial design showed that corn steep liquor has a positive outcome, which our study also shows, while the oil residue showed a negative effect, unlike in our study. According to Albuquerque et al. [21], the factorial design showed the influence of urea, ammonium sulfate, dihydrogen potassium phosphate and corn oil when bioemulsifier is produced by *Candida lipolytica* (UCP 0988), and they also observed that corn oil and KH_2_PO_4_(NH_4_)_2_SO_4_ had a positive effect on the emulsification activity. Positive results were obtained with corn steep liquor and waste soybean oil on biosurfactant production by *Candida lipolytica*.

In this study, the data obtained are promising given that the biotechnological production of bioemulsifier depends on developing viable processes that reduce production costs, bearing in mind that currently the end cost of the culture medium is around 50% of total production costs [16,22].

### 3.2. Characterizing Droplets with Regard to Stable Emulsions

Comparing the distribution of the size of the droplets and the emulsification index (E24) showed that at the beginning of biosurfactant production, the droplets varied in size and this fact influenced the stability of the emulsion. Dickinson et al. [12] have studied experimentally the coalescence of millimeter-sized oil drops at an oil/water interface and observed the larger droplets burst faster than the smaller ones. The authors described that droplets size range depending of the coalescence behavior with the deformability of the drop surface.

In this work, we suggested the factor responsible for the stability of the droplet size was the high concentration of bioemulsifier produced by *Candida lipolytica* after 96 h of cultivation, which resulted in stable drops with dimensions 0.5 to 10 μm, and activity of 6.794 UAE. The bioemulsifier was constituted by carbohydrate (37.62%), lipids (12.2%), and protein (50.18%).

According to Langenvin [23], the higher concentration of the bioemulsifier on free metabolic liquid shown greater stability of the droplets formed and the efficiency of the emulsification is closely related to the adsorption of surface-active molecules.

Cirigliano and Carmam [6] reported that the stability of the water/oil emulsions depends on the levels of various properties of the bioemulsifier, including temperature, sodium and potassium ion content and pH, being maintained. However, Camacho-Chab et al. [24] and Gudina et al. [25] described that the high molecular weight bioemulsifier is responsible for maintaining the stability of emulsions.

Recently, Rufino et al. [8] used the same yeast *C. lipolytica* and industrial waste from vegetable oil refinery as substrate and produced a biosurfactant constituted by protein (50%), lipids 20% (20%), and carbohydrate (8%).

According to Jillavenkatesa et al. [26], the analysis of the emulsion requires two important parameters: the size of the drop of the dispersed phase and its concentration. These factors influence the viscosity, stability and coalescence. According to Langervin [23], a stable emulsion enables its droplets to be retained and dispersed in the continuous phase for a considerable time, without phase separation, while also bearing in mind that it is higher concentrations of bioemulsifier that ensure that stability is increased and the coalescence of drops is reduced.

The complexes are formed between anionic polysaccharides and proteins at pH values below the protein isoelectric point where they carry a net positive charge. One approach, first reported by Bungeberg de Jong [27], is to use polysaccharide–protein coacervates as emulsifiers [28] but more recent interest has been given to using soluble electrostatic complexes [29,30]. A second approach is to form an emulsion using a protein as the emulsifier and then add the polysaccharide which adsorbs onto the protein layer to form a secondary layer on top [31].

### 3.3. Stability of Bioemulsifier

Several factors such as salinity, pH and temperature affect the activity of bioemulsifiers [16,19]. These results were similar to those obtained by Santos et al. [19] who obtained 90% emulsification in acid pH. The capacity of the bioemulsifier to maintain its stability after it has been subjected to high temperatures is an important characteristic, which industries that require their products to be sterilized at high temperatures should be aware of [19]. In this context, the bioemulsifier produced by *Candida lipolytica* in this study proved to be stable at all temperatures tested. Rufino et al. [32], demonstrated loss of emulsifying stability by biosurfactant produced by *Candida lipolytica* at 100 °C.

### 3.4. Potential of Bioemulsifier on Displacement of Hydrophobic Compound in Seawater

The oil dispersion test is a sensitive and convenient assay system to examine the presence of biosurfactants/bioemulsifiers by identifying a clear area on the oil surface [28].

The biosurfactant produced by *Cyberlindnera samutprakarnensis* (JP 52) yeast grown in glycerol obtained an activity of 44.5 [29]. In the study by Silva et al. [30], the bioemulsifier produced by *Cunninghamella echinulata* in waste soybean oil and corn steep liquor showed that the area of oil dispersion was 37.36 cm^2^.

### 3.5. Application of Bioemulsifier to Remove a Hydrophobic Compound

Washing contaminated soils with aqueous solutions solubilizes the adsorbed pollutants in the soil. Not only removing water but also adding surfactants, organic or inorganic acids, sodium hydroxide, etc., increases the efficiency of the removal process [31].

Andrade et al. [33], using this biosurfactant produced by the metabolic liquid *Candida glabrata*, used the washing method to remove 95.7% of burnt engine oil. The results obtained by Santos et al. [19] were similar to those in our study, the biosurfactant produced by *C. lipolytica* UCP0988 grown in animal fat and 92.3% corn steep liquor removed burnt engine oil from sand.

According to Urum et al. [34], the longer the oil is in contact with sand, the more it will be adsorped from this surface. Therefore, molecules of bioemulsifier/biosurfactant are less efficient at removing diesel oil, because it is difficult for biomolecules to interact with oil.

Results reported in the literature show that the biosurfactant of *C. antarctica* removes about 50% of the oil adsorbed in the sand [35]. Biosurfactant produced by *Bacillus* species removed about 30% of the oil contained in a packed column [35].

### 3.6. Production of Biomass and Total Lipids by Candida lipolytica UCP 0988 Using Agro-Industrial Waste

In our study, we observed that the maximum concentrations of corn steep liquor and waste soybean oil in the production medium favored a high production of biomass.

The data demonstrate that corn steep liquor was the most significant variable. In the study by Daverey and Pakshirajam [36], the biomass of *Candida bombicola* was reduced when grown only in carbon sources (oil and molasses). The researchers concluded that the absence of a nitrogen source in the culture medium caused the low growth of yeast. Santos et al. [19] concluded that corn steep liquor was sufficient as a nitrogen source for the growth of *Candida lipolytica*. This study shows that the culture medium containing waste corn steep liquor and soybean oil can replace synthetic culture media for the growth of *Candida lipolytica*. Several studies have shown the ability of *C. lipolytica* to grow in hydrophobic media, which is due to its evolving genes that encode surfactants [8,37].

*C. lipolytica* is a model microorganism used to produce lipid metabolizing hydrophobic substrates [5,37]. As shown in Table 3, in this study, *C. lipolytica* was able to produce lipids which had 0.425 g/g biomass, corresponding to a 42.5% of accumulation, thus indicating that these residues are promising for the production of lipids, as they contribute to reducing costs in the bioprocess and waste has added value.

Similar results were obtained by Papanikolaou and Aggelis [38] when using industrial glycerol, as well as Areesirisuk et al. [39] when using hydrophilic carbon source. On the other hand, *C. lipolytica* showed the suitability molasses at 8% and increased the lipid production to 59.9%; however, molasses contains heavy metals [40].

However, the use of crude glycerol substrates (10%) and Tween 20 (1%) caused lower lipids production [41]. The use of crude glycerol at 10% showed 40.6% of total lipids production by *Trichosporonoides spathulata* [42].

Galafassi et al. [43] using hydrophilic substrates as corn steep solids 2% and Yeast extract (0.1%) produced 52.1% of lipids. *Cryptococcus curvatus* using accetate (15.9%) as hydrophilic carbon source produced higher level of total lipids [44].

To obtain the maximum accumulation of lipids by this microorganism in a hydrophobic substrate at maximum concentration and accumulation, we compared total lipids produced by different yeasts using diverse substrates (Table 5).

According to Beopoulos et al. [37], this route involves the absorption of fatty acids, oils and triacylglycerides from the culture medium. Figure 5 shows the accumulation of total lipid content into cells from 0 to 96 h in Assay 4 (5% corn steep liquor and 8% waste soybean oil) by cytochemical analysis using Sudan Black staining, thereby revealing the presence of lipid bodies of dark color. Burdon [45] first described the staining of lipid bodies in fungi using the dye Sudan Black. This has been recently used to analyze these compounds in cells as a qualitative analysis in order to investigate oleaginous microorganisms [46,47].

Our results were higher than those given in the studies by André et al. [48], while Makri et al. [48] obtained 30.7% and 22.3%, respectively, total lipids by the yeast grown in glycerol. Previous studies have stated that when the yeast is grown on hydrophobic substrates, the accumulation of lipids is not greater when compared to the hydrophobic substrates. Bati et al. [49], cultivating the yeast corn oil, obtained a percentage of 55% of total lipids. Papanikolaou et al. [40], using animal fat as a substrate in the culture medium, obtained approximately 44% total lipid by yeast.

El Bialy et al. [50] obtained good results from cultivating *Candida lipolytica* using waste oil, which they obtained from fast food outlets, as a nutritional source. When the oil was obtained after it had been used to fry vegetables, the yeast accumulated 57.89% of lipids and when cultivated in other waste oils such as those used to fry fish and meat, the total lipids obtained were 45.49% and 34.02%, respectively.

### 3.7. Fatty Acid Profile of Total Lipids

Papanikolaou et al. [40] obtained a similar profile to that of our study after cultivating *Candida lipolytica* in glycerol. This produced 47% oleic acid, 21% linoleic acid, 15% palmitic acid, 11% stearic acid and 2% palmitoleic acid. According to Islam et al. [51], there is no single fatty acid that accounts for the property of the fuel. Therefore, what is important is the presence of saturated fatty acids that favors the ignition quality. The excess saturated fatty acids lead to clogging due to the product freezing, whereas the presence of monounsaturated and polyunsaturated fatty acids, such as linoleic and oleic acid, which present a melting point of −5 and 4 °C, respectively, complicates the freezing of biodiesel.

It is worth giving importance to the significant presence of polyunsaturated fatty acids in biodiesel, although they are difficult to freeze due to their lower melting points. This reduces the cetane number and the stability of oxidation, which is undesirable for biodiesel [52]. Oxidation causes an increase in viscosity and further clogging of filters due to insoluble materials being produced, and the increase of the acidity and the presence of hydroperoxide that cause corrosion of the injection system [53].

In this study, the profile of FAMEs obtained from the biomass of *C. lipolytica*, shows up next to the soybean oil profile. Soybean oil is a major raw material for biodiesel production in Brazil, the United States and Europe and consists of linoleic acid (about 54%) and oleic acid (approximately 22%) and other saturated fatty acids [54]. However, compared to the profile of fatty acids produced by *C. lipolytica*, linoleic acid is dominant in soybean oil and the significant presence of this fatty acid, as well as of other polyunsaturated fatty acids, increases iodine values in biodiesel. Due to the presence of these unsaturated fatty acids, soybean oil has an iodine value of 129.7 (g of I_2_/100 g); the European Union limits the amount to 120 mg of I_2_/g, but the USA does not specify iodine values as a quality parameter of biodiesel [54,55].

### 3.8. Transesterification/Direct Esterification of Biomass

The simultaneous method of extraction and transesterification/esterification of biomass ensures the complete reaction of lipids in methyl esters of fatty acids. This includes free fatty acids being present in the total lipids, which is an important factor that determines the acidity of biodiesel. In addition, in acid catalysis, soap is not formed while alkaline catalysis has low neutralization of free fatty acids [55,56,57]. Many studies have used this method with methanol and chloroform as a solvent, as well as sulfuric acid (H_2_SO_4_), hydrochloric acid (HCl), and boron trifluoride (BF_3_) as acid catalysts [55].

In this study, approximately 96.70% of biodiesel was obtained from total lipids that were transesterified. Only saponifiable lipids (triacylglycerides, dialcilglicerídeos, monoalcilglicerídeos and polar lipids) and free fatty acids are converted. These values indicate that the method used for transesterification/esterification by acid catalysis is significant in comparison with traditional methods that extract lipids [58,59].

## 4. Materials and Methods

### 4.1. Microorganism

The yeast *Candida lipolytica* UCP 0988 was kindly provided by the Culture Collection UCP (Universidade Católica de Pernambuco)-Catholic University of Pernambuco, Recife-PE, Brazil, and registered in World Federation in Culture for Collection (WFCC). The yeast was maintained in an anamorphous state at 5 °C in glass tubes with yeast culture medium on a slant with an inclined plane (YMA (Yeast Malt Agar), Sigma Y-3127, Sigma-Aldrich Co., St. Louis, MO, USA), Transfers were made periodically to fresh agar slants to maintain the viability of the microorganism.

### 4.2. Substrates

Two types of industrial waste were used as substrates to produce the biosurfactant. Corn steep liquor was obtained from Ingredion Brasil Ing. Ind. Ltda, Cabo de Santo Agostinho, Pernambuco, Brazil and soybean oil residue, provided by informal commerce. The soybean oil residue was used as the main carbon source and the corn steep liquor was used as the nitrogen source. Both agro-industrial by-products also provided other nutrients that are essential for the metabolism of yeast.

### 4.3. Growth Conditions

*C. lipolytica* was grown in 250 mL Erlenmeyer flasks, containing 100 mL of medium consisting of corn steep liquor and residual soybean oil in accordance with the concentrations established by 2^2^ factorial designs as shown in Table 1. All media were added to a base containing salts NH_4_NO_3_ (0.1%), KH_2_PO_4_ (0.02 g·L^−1^) and MgSO_4_ (0.02 g·L^−1^). The pH of the production medium was adjusted to 6.0 by adding 1 M NaOH or 1 M HCl solution and the inoculum was 1% of the culture containing 10^7^ cel·mL. The bottles were kept under orbital agitation at 50 rpm, and incubated for 96 h at 28 °C. After this period, the culture was centrifuged at 10,000× *g* followed by filtration through Millipore 0.45 µm filter (Lakeside Drive, Foster City, CA, USA), in order to separate the cellular metabolic liquid. The metabolic liquid was subjected to testing activity and emulsification index.

### 4.4. Determining the Production of Bioemulsifier

To investigate the production of bioemulsifier, emulsification activity was performed in accordance with the methodology described by Cirigliano and Carman [6] with the results expressed in Emulsifying Activity Units (EAU) and as an emulsifying index (E_24_) following the method of Cooper and Goldenberg [60] and the results is expressed in percentages (%). Aliquots were collected after every 24 h of fermentation, filtered through Millipore 0.45 µm filter (Lakeside Drive, Foster City, CA, USA), and centrifuged at 4000 rpm for 20 min so as to obtain cell free metabolic liquid. The hydrophobic substrates used for the activity test and emulsifying index were n-hexadecane, corn oil and crude oil.

### 4.5. Isolating the Bioemulsifier

The cell-free culture broth was precipitated with two volumes of alcohol 70%. After 24 h at 4 °C, samples were centrifuged at 5000× *g* for 30 min, washed twice with cold water and dried at 37 °C for 24 to 48 h.

### 4.6. Characterizing the Bioemulsifier

The concentration of protein in the biosurfactant isolate was estimated by using the total protein test kit from Labtest Diagnóstica S.A., Brazil. The total carbohydrate content was estimated by the phenol-sulfuric acid method [61]. The lipid content was determined according to Manocha et al. [62]: 0.5 g of the isolated material was extracted with chloroform:methanol in different proportions (1:1 and 1:2, *v*/*v*). The organic extracts were then evaporated under vacuum and the lipid content determined by gravimetric estimation.

### 4.7. Determining the Ionic Character of the Bioemulsifier

The ionic charge of the biosurfactant was determined by using a Zeta potentiometer, model ZM3-D-G, Zeta Meter System 3.0+, with direct images to the video of the Zeta Meter, San Francisco, CA, USA.

### 4.8. Measuring the Droplets

Emulsions were prepared with 2 mL of engine oil, 1 mL of cell-free metabolic broth (0–24, 24–48, 48–72 and 96 h of fermentation from Assay 4) and the anionic surfactant sodium dodecyl sulfate (SDS) was used as a positive control. The diameter of the droplets obtained from the emulsion were observed and measured using a light microscope (Olympus BX50) with an increase of 100× and a digital camera was used to capture the images.

### 4.9. Oil Spreading Test

The biosurfactant produced was separated from culture media by centrifuging so as to obtain culture supernatant and this was characterized by measuring the diameters of clear zones caused when a drop of biosurfactant containing the solution was placed on the oil–water surface. Fifty milliliters of distilled water were added to a large Petri dish (15 cm in diameter) followed by adding 20 µL of crude oil to the water surface and 10 µL of culture broth supernatant. A clear halo was visible under light. The area of this circle was measured and calculated to determine the oil displacement area (ODA) using the following equation:

(1) $$\mathrm{ODA}\;=\frac{22}{7}\;{\left(\mathrm{radius}\right)}^{2}{\;\mathrm{cm}}^{2}$$

The triplicate assays from the same sample were measured as in Techaoei et al. [63].

### 4.10. Stability Studies

Stability studies were conducted using the cell-free broth obtained by centrifuging the cultures at 5000× *g* for 20 min. Forty milliliters of the cell-free culture broth were heated at 50 and 100 °C, and cooled to room temperature, after which the emulsification index was determined. The pH of the cell-free broth was adjusted to different values (2.0–10.0) so as to determine stability. The effect of adding NaCl (at concentrations of 2.0%–20%) was also determined. All assays were carried out in triplicate.

### 4.11. Applying Bioemulsifier to Remove Diesel Oil from Contaminated Sand

The suitability of the biosurfactant for enhanced oil recovery was determined using 10.0 g of sand impregnated with 1.0 g of diesel oil for 7 days at room temperature. The biosurfactant produced by *C. lipolytica* was used in the removal tests. Fractions of contaminated sand were transferred to 125 mL Erlenmeyer flasks, followed by adding 20 mL of the cell-free broth or 20.0 mL of distilled water (control). The samples were incubated in a rotary shaker (150 rpm) for 24 h at 30 °C and centrifuged at 5000× *g* for 10 min to separate the laundering solution and sand. The amount of diesel oil in the sand after contact with the biosurfactant was gravimetrically determined as being the amount of material extracted from the sand by hexane [31].

### 4.12. Determining the Biomass

To determine the biomass, 50 mL aliquots of the production medium were withdrawn at time intervals from 24 to 96 h, and centrifuged at 10,000× *g* at 28 °C for 20 min to obtain biomass. Thereafter, the biomass was washed with distilled water, lyophilized and kept in a desiccator until constant weight.

### 4.13. Cytochemical Analysis of Lipids

A cytochemical analysis of lipids present in *C. lipolytica* was performed in accordance with modifications to the method of Sheehan and Storey [64]. The cell samples were fixed in glutaraldehyde and then washed in phosphate buffered saline (PBS). They were then immersed in Sudan Black B stain for 10 min in the dark. After this step, the samples were rinsed in 70% alcohol to remove excess dye, and then washed with distilled water, counter-stained with safranin for 30 s and washed again with PBS. The slides were viewed under an optical microscope using a 100× lens. The oil droplets present in the yeast cells were stained in black or dark blue.

### 4.14. Lipid Extraction

The lyophilized biomass (1.0 g) was extracted with 20 vol. each of chloroform/methanol as 2:1, 1:1 and 1:2 (*v*/*v*) mixtures. The cultures were suspended in chloroform/methanol (2:1 *v*/*v*) and homogenized for 1 h. The extract was separated by centrifuging at 5000× *g* for 10 min. The supernatant was dried to constant weight under vacuum in a desiccator [62].

The percentage of total lipids present in the biomass was quantified by gravimetric method using the following equation:

(2) $$\mathrm{Total}\;\mathrm{lipids}\;\left(\%\right)=\frac{\mathrm{Lipids}}{\mathrm{Dried}\;\mathrm{biomass}\;}\times 100$$

### 4.15. Identifying Fatty Acids from Biomass

The fatty acids of the biomass were identified and quantified using a Varian gas chromatograph fitted with an HP-5 fused with an ionization detector and an automatic injector of silica (5% diphenyl and 95% dimethylpolysiloxane) capillary column 0.25 mm × 30 m. The initial temperature of the column oven heating ramp was 150 °C and the final temperature was 250 °C. The temperature of the injector and detector was 280 °C, with helium was used as a carrier gas. The biomass was methylated in esters of fatty acids in accordance with Durham and Kloos [65].

### 4.16. Transesterification/Esterification

For the direct conversion of biomass into biodiesel, the method of Vicente et al. [55] was used. The biomass was trans esterified/esterified for 8 h at 65 °C at 900 rpm with a boron triofluoride solution as the acid catalyst and 10:1 methanol/chloroform. Water and hexane were added to the mixture, which was then centrifuged and the solvent was removed by rotary evaporator. The yield of biodiesel produced was calculated by gravimetry.

### 4.17. Factorial Design

Factorial design (2^2^) with eight assays and four replicates at the central point were carried out to investigate the main effects and interactions of the independent variables of corn steep liquor (%) and waste soybean oil (%) on the response of variable production to bioemulsifier and lipids by *Candida lipolytica* where the concentrations of the independent variables were: corn steep liquor 1% (Level −1), 3% (Level 0), and 5% (Level +1) and waste soybean oil 2% (Level −1), 5% (Level 0), and 8% (Level +1).

## 5. Conclusions

The production of bioemulsifier and biodiesel by *Candida lipolytica* UCP 0988 depends on the raw material used. This study used waste soybean oil as carbon source and corn steep liquor as nitrogen nutrient for growth and secondary metabolites. The best bioemulsifier productivity was achieved when the assay was conducted with a low level of nitrogen and sources high in carbon (Assay 4). In this condition, the crude bioemulsifier gave high emulsifying activities against engine burned motor oil, and homogeneous, compact, smaller and stable droplets, and higher units of emulsification activity (UEA). Additionally, *C. lipolytica* produced important lipids for biodiesel production. The properties of the bioemulsifier terms showed stable emulsion against temperature, pH and salinity. The bioemulsifier and biodiesel produced by the conversion of organic residues make the bioprocess economically viable and this suggests these may be used for further environmental or industrial applications.

## Figures and Tables

**Table 1:** Bioemulsifier production by *C. lipolytica* using factorial design.

Assays	Emulsification Index (%)			Emulsification Activity (UEA)
	Burned Engine Oil	*n*-Hexadecane	Corn Oil	*n*-Hexadecane
1	64.00	Negative	35.00	0.945
2	91.40	14.70	50.00	5.693
3	93.33	60.00	55.00	6.281
4	96.66	63.63	58.00	6.704
5	66.66	Negative	50.00	4.233
6	70.96	Negative	47.00	4.335
7	72.85	Negative	50.00	4.370
8	74.28	Negative	50.00	4.379

Independent variables : (−1): corn steep liquor 1% (*v*/*v*) and 2% (*v*/*v*) of waste soybean oil; Central point (0): 3% (*v*/*v*) corn steep liquor and 5% (*v*/*v*) of waste soybean oil; (+1): 5% (*v*/*v*) corn steep liquor and 8% (*v*/*v*) of waste soybean oil.

**Table 2:** Evaluation of the bioemulsifier produced by *Candida lipolytica* properties regarding droplet size, Emulsification index (E24), Units of Emulsification Activity (UEA) and characterization of emulsification *.

Time of Growth (h)	Droplet Size Measurement (µm)	Emulsification Index (E24 (%))	Units of Emulsification Activity (UEA)	Emulsification Properties
**Liquid Medium (Control)**	1–30	Not formed	0.018	No emulsification. Drops without globular form and large size. Droplets unstable.
**24**	0.5–10	28.57	0.891	Turbid with sedimentation, globular droplets and large flocculation. Droplets unstable.
**48**	0.3–10	47.06	5.648	Homogeneous with smaller and large oil droplets. Droplets unstable.
**72**	0.1–10	76.47	5.812	Droplets were homogeneous, smaller and stable.
**96**	0.1–5	96.66	6.101	Droplets were homogeneous, compact, smaller and stable.
**SDS (Control)**	2.5–12	100	6.933	Droplets were homogeneous, compact smaller and stable.

* Assay 4 (5% (*v*/*v*) corn steep liquor and 8% (*v*/*v*) waste soybean oil).

**Table 3:** Production of biomass and total lipids by *Candida lipolytica* using 2^2^ factorial design.

Assays	pH	Biomass (g/L)	Lipids (g/L Biomass)	Lipids (g/g Biomass)
**1**	4.1	1.96	0.696	0.355
**2**	4.2	4.41	1.87	0.425
**3**	4.1	10.02	3.15	0.314
**4**	4.2	12.71	4.96	0.390
**5**	4.1	6.71	2.17	0.323
**6**	4.1	6.25	2.25	0.360
**7**	4.2	6.73	2.47	0.367
**8**	4.2	7.16	2.20	0.307

Independent variables : (−1): corn steep liquor 1% (*v*/*v*) and 2% (*v*/*v*) of waste soybean oil; Central point (0): 3% ( *v*/*v*) corn steep liquor and 5% (*v*/*v*) of waste soybean oil; (+1): 5% (*v*/*v*) corn steep liquor and 8% (*v*/*v*) of waste soybean oil.

**Table 4:** Fatty acid profile of total lipids from biomass of *Candida lipolytica* in Assay 4 after 96 h.

Fatty Acid Methyl Esters (%)	
Capric acid (C10:0)	–
Myristic acid (C14:0)	–
Palmitic acid (C16:0)	28.4
Palmitoleic acid (C16:1)	–
Stearic acid (C18:0)	7.7
Oleic acid (C18:1)	42.8
Linoleic acid (C18:2)	19.0
α-Linolenic acid (C18:3–ω-3)	–
γ-Linolenic acid (C18:3–ω-6)	2.1
Arachidic acid (C20:0)	–
Gadoleic acid (C20:1)	–
Behenic acid (C22:0)	–

Not detected: –.

**Table 5:** Comparison of lipid production by yeasts using different substrates with *Candida lipolytica* UCP 0988 in this study.

Microorganism	Carbon Source (Substrates)	Lipids (%)	References
*Candida lipolytica*	Waste soybean oil (8%)	42.5	Present study
*Candida lipolytica*	Molasses at 8%	59.9	Karatay and Donmez [40]
*Yarrowia lipolytica*	Industrial glycerol at 50%	43.0	Papanikolaou and Aggelis [38]
*Cryptococcus curvatus*	Acetate (15.9%)	73.4	Gong et al. [44]
*Rhodotorula glutinis*	Crude glycerol 10% and Tween 20 at 1%	35.2	Saenge et al. [41]
*Rhodotorula graminis*	Corn steep solids 2% and Yeast extract (0.1%)	52.1	Galafassi et al. [43]
*Trichosporonoides spathulata*	Crude glycerol at 10%	40.6	Kitcha and Cheirsilp [42]
*Pseudozyma parantarctica*	Glucose (40%)	43.5	Areesirisuk et al. [39]
